# Shorter Telomeres Related to Posttraumatic Stress Disorder Re-experiencing Symptoms in Sexually Assaulted Civilian Women

**DOI:** 10.3389/fpsyt.2022.835783

**Published:** 2022-05-19

**Authors:** Carolina Muniz Carvalho, Bruno Messina Coimbra, Gabriela Xavier, Amanda V. G. Bugiga, Tamiris Fonseca, Miranda Olff, Renato Polimanti, Andrea Feijó Mello, Vanessa Kiyomi Ota, Marcelo Feijó Mello, Sintia Iole Belangero

**Affiliations:** ^1^Department of Psychiatry, Universidade Federal de São Paulo, São Paulo, Brazil; ^2^LiNC - Laboratory of Integrative Neuroscience, Universidade Federal de São Paulo, São Paulo, Brazil; ^3^Department of Psychiatry, University of Amsterdam, Amsterdam Public Health Research Institute and Amsterdam Neuroscience Research Institute, Amsterdam, Netherlands; ^4^Genetics Division of Department of Morphology, Genetics of Universidade Federal de São Paulo, São Paulo, Brazil; ^5^ARQ National Psychotrauma Centre, Diemen, Netherlands; ^6^Department of Psychiatry, Yale School of Medicine, VA CT Healthcare Center, West Haven, CT, United States

**Keywords:** cellular aging, telomere–genetics, PTSD symptom clusters, sexual assault against woman, PTSD–posttraumatic stress disorder

## Abstract

Telomeres are short tandem repeats of “TTAGGG” that protect the chromosome ends from deterioration or fusion of chromosomes. Their repeat length shortens with cell division acting as a biomarker of cellular aging. Traumatic stress events during adulthood or childhood have been associated with posttraumatic stress disorder (PTSD) and short leukocyte telomere length (LTL). This study investigated whether LTL was associated with PTSD in a Brazilian sample of sexually assaulted civilian women at two time points: baseline and 1-year follow-up. At baseline, we assessed 64 women with PTSD following sexual assault (cases) and 60 women with no previous history of sexual trauma or mental disorders (healthy controls – HC). At follow-up visit, 13 persistent PTSD cases, 11 HCs, and 11 PTSD remitters patients were evaluated. PTSD diagnosis and severity were assessed using Mini International Neuropsychiatric Interview (Diagnostic and Statistical Manual of Mental Disorders III/IV criteria) and Clinician-Administered PTSD Scale for DSM-5 (CAPS-5), respectively. LTL was measured using multiplex real-time polymerase chain reaction (PCR). In the baseline analysis, we observed that LTL was associated with re-experiencing symptoms (*B* = −0.16; confidence interval (CI) 95% = −0.027—−0.005; Bonferroni-adjusted *p*-value = 0.02), but no association was observed between other PTSD symptoms and LTL. In the longitudinal analysis, telomere shortening was no longer observed in patients with PTSD and PTSD remitters. In conclusion, our findings indicate that shorter baseline LTL is associated with early stage of PTSD re-experiencing symptoms in recently sexually assaulted women.

## Introduction

Telomeres are short tandem repetitions of the non-coding sequence TTAGGG (1) in the final portion of chromosomes and act as protective structures of the chromosome to maintain genomic stability (1, 2). Their repeat length shortens with cell division (3), possibly because of incomplete replication of the telomeric extremities, recombination, epigenetic regulation, cumulative exposure to oxidative stress, and stress hormones (2, 4). Telomere shortening over time establishes telomere length (TL) as a valuable marker of cellular aging (1). Trauma-induced stress has been associated with telomere shortening (4, 5) among individuals with posttraumatic stress disorder (PTSD) (6).

Posttraumatic stress disorder may occur after experiencing or witnessing a traumatic event and is characterized by four symptom clusters: re-experiencing, avoidance, hyperarousal, and negative alterations in cognitions and mood (7). The core symptoms of PTSD may lead to an overreaction of biological systems, including functions related to telomere length maintenance such as antioxidant defense and well-being (8). Moreover, this complex clinical condition is often associated with the onset of age-related diseases, e.g., cardiovascular, neurodegenerative, and inflammatory diseases, and early mortality (8, 9), suggesting that health decline in PTSD may be related to shorter TL. Previous findings suggest that repeated and prolonged activation of the stress response systems after trauma exposure may be one of the factors that contribute to cellular senescence in patients with PTSD (6, 10–17). Furthermore, trauma response and elevated psychological stress in PTSD may be implicated in telomere shortening through high inflammatory activity, increased sympathetic nervous system activation, and dysregulation of the hypothalamic–pituitary–adrenal (HPA) axis (5, 18, 19).

Despite the increasing number of evidence of the association between telomere shortening and PTSD diagnosis (6, 10–14), there is still less research on the relationship between PTSD and TL in traumatized civilian women. Also, most studies on PTSD-related TL enrolled samples of chronic patients. Thus, we hypothesized that women who developed PTSD following exposure to a recent sexual assault (1 to 6 months before PTSD and TL assessment) would have shorter TL than healthy controls with no history of sexual trauma, through pathways and stress reactions related to trauma experience possibly related to cellular damage. We further hypothesized that severe PTSD symptoms related to brain hyperactivity, such as increased re-experiencing of recollections related to the traumatic event (i.e., flashbacks, nightmares, thoughts, and intrusive memories) and hyperarousal (i.e., hypervigilance, physiological reactivity, and sleep disturbances), expose the individual to psychological stress that may have a weathering effect on LTL.

Thereby, we aimed to investigate whether there are signs of accelerated telomere shortening in a Brazilian cohort of women who developed PTSD diagnosis after a recent sexual assault, using a cross-sectional and longitudinal design. We also explored the link between TL and PTSD symptom clusters. Further, increased comorbid alcohol use is commonly observed in patients with PTSD (20, 21), and research findings suggest that alcohol abuse is associated with shorter TL, possibly due to alterations in oxidative stress and inflammation (22–24); thus, we investigated the effects of alcohol use on TL in this cohort. We also examined the confounding effect of social deprivation and lower educational attainment, as the psychosocial stressful situations encountered by individuals with a low socioeconomic status have been reported in the literature to influence telomere decline (25–27). Then, to better understand the association between PTSD and TL, we considered the confounder effect related to alcohol use and social deprivation, and lower educational attainment.

## Materials and Methods

### Study Population

In this longitudinal study, clinical assessments and blood samples were obtained at two time points: baseline and 1-year follow-up. At baseline, we recruited 63 women with a positive PTSD diagnosis who were the victims of sexual assault from 1 to 6 months before study inclusion (case group), and 60 women with no history of sexual trauma or mental disorders (healthy control – HC group). The longitudinal sample was comprised of 24 patients with PTSD and 11 HC participants who returned for follow-up assessments. Of the 24 patients with PTSD who completed the 1-year follow-up, 13 were classified as having persistent PTSD and 11 as being PTSD remitters based on the Clinician-Administered PTSD Scale for DSM-5 (CAPS-5).

This study is the part of a larger study on neuroprogression in the early stages of PTSD that has been described in detail elsewhere (28). The Research Ethics Committee of Universidade Federal de São Paulo (UNIFESP) approved the study protocol, and all participants provided written informed consent.

### Selection and Recruitment of Participants

Eligible women for the case and HC group were those aged 18–45 years. Women in the case group and HC that were undergoing psychiatric or psychological treatment were excluded. Other exclusion factors were having a sexually transmissible disease, unstable medical conditions, neurological disorders, schizophrenia, bipolar disorder, current use of corticosteroid medication, pregnancy, and menopausal symptoms. Participants included in the case group were recruited at Hospital Pérola Byington, a women’s specialized health center that provides gynecological care for the victims of sexual assault in São Paulo, Brazil. Participants classified as HC were recruited in the community through social media and advertisement. Participation in the study was voluntary and no financial compensation was offered.

### Clinical Assessments

Psychiatric disorder diagnoses were obtained using the Mini International Neuropsychiatric Interview (Diagnostic and Statistical Manual of Mental Disorders III/IV criteria) in all participants (29) in both visits: baseline and follow-up. PTSD diagnosis was assessed in patients in both visits by the CAPS-5. CAPS-5 is the gold standard scale for assessing PTSD diagnostic status and symptom severity. It comprises 30 items that investigate the frequency and intensity of PTSD symptoms based on four symptom clusters related to PTSD diagnosis: avoidance, re-experiencing, negative alterations in cognitions and mood, and hyperarousal (30, 31). In addition, alcohol consumption over the past year was investigated at baseline for all participants using the Alcohol Use Disorders Identification Test (AUDIT) to identify early signs of hazardous drinking and dependence (32).

### Sociodemographic Evaluation

We developed a sociodemographic inventory to collect baseline relevant sociodemographic characteristics of all participants, such as age, education, and income. Self-reported information regarding the participants’ per-person income in Brazilian real (BRL) (1.00 BRL approximately 0.25 USD) was collected using the standard questionnaire, only at baseline assessments. Educational attainment was referred to in our study as the highest level of education the participant has completed at the time of the first assessment of this study. Based on the participants’ years of study, educational attainment was categorized into three categories: less than 4 years of study (primary school incomplete - N_case_ = 0; N_HC_ = 1), 4 to 12 years of study (primary school and high school - N_case_ = 34; N_HC_ = 4), and over 12 years of study (Bachelor’s degree or more - N_case_ = 29; N_HC_ = 54).

### Telomere Measurement

Approximately 10 ml of blood in EDTA tubes (Becton Dickinson, United States) of each participant was collected for DNA extraction and telomere measurement in both visits: baseline and follow-up. According to the manufacturer’s protocol, DNA was extracted using the Gentra Puregene Kit (Qiagen, United States). Leukocyte TL (LTL) was measured in triplicate wells following the protocol developed by Cawthon et al. (33) using multiplex real-time polymerase chain reaction (qPCR) to estimate the relative T/S ratio between the telomeric region copy number (T) and a single-copy gene (S, the albumin gene). T/S ratio is proportional to the mean LTL in the peripheral blood. Further, a positive control sample was used in all plates, as a quality control inter-assay, being that the inter-assay coefficient of variation for this study was 7.5%.

### Statistical Analyses

Clinical and sociodemographic characteristics assessed in the first visit (baseline), i.e., age, per-person income, and alcohol consumption (AUDIT score), were compared between case-HC groups using independent sample *t*-tests, and Pearson’s chi-square (χ2) was applied to assess the educational attainment category difference between groups. In addition, Spearman’s correlations were used to assess the baseline correlations between per-person income and PTSD symptoms, and between per-person income and harmful alcohol use. These analyses identified the baseline association between clinical and sociodemographic measures that could potentially confound the relationship between PTSD and LTL.

The normality of LTL data was evaluated by Shapiro–Wilk (SW) tests using cross-sectional and longitudinal data. Primary analyses using linear regression models were conducted to investigate the effect of age on LTL measured at baseline and follow-up time. We used the unstandardized residual values of this regression as the predictor variable of relative LTL (called relative LTL adjusted by age) for all secondary analyses and Pearson’s correlations to avoid bias related to age in the relative TL.

First, Pearson’s correlations were applied to investigate the baseline effects of per-person income on relative LTL adjusted by age in the case and HC groups. Also, Pearson’s correlations tested the baseline correlation between harmful alcohol use and relative LTL adjusted by age in all participants. Secondary analyses consist of a series of regressions models (logistic or linear regressions) to evaluate the effect of PTSD assessed at all time points, including PTSD diagnosis and PTSD symptoms on relative LTL adjusted by age, controlling these associations for potentially confounding baseline measures.

In the baseline analysis, logistic regression evaluated the association of relative LTL with trauma exposure (i.e., sexual assault) and evaluated whether shorter telomeres were associated with PTSD status compared to HC group, adjusting this model for alcohol dependence (AUDIT score), educational attainment, and per-person income. Linear regression adjusted by per-person income verified whether PTSD symptoms based on four clusters of CAPS-5 could predict the relative LTL using the baseline data. The partial regression plot was used for visualization of these linear model’s results and reflects the scatter of partial correlation: the plot shows the residuals of relative LTL adjusted by age (i.e., unstandardized residuals of LTL) and per-person income vs. residuals of the predictor variable (i.e., PTSD status) on the dependent variable.

In the follow-up analysis, logistic and linear regressions were conducted to investigate, respectively, whether relative LTL was associated with PTSD status (persistent PTSD case, PTSD remitter, and HC) or whether PTSD symptoms could predict shorter relative LTL.

Finally, we performed *post hoc* power analysis using the function *pwr.f2.test* of R package *“pwr” - basic functions for power analysis* to calculate the power for regression models which are statistically significant.

All statistical analyses were performed using the Statistical Package for the Social Sciences (SPSS) version 21 and RStudio version 1.4.1103. Bonferroni correction was applied to adjust the significance threshold accounting for the number of regressions performed, and the level of significance was set at 0.05.

## Results

### Participants’ Characteristics

Table 1 summarizes the mean difference in baseline sociodemographic characteristics and alcohol use in 63 cases and 60 HCs. The groups significantly differed in age, education, and alcohol consumption. The case group was younger (*p* = 0.003), had lower per-person income (*p* = 0.002), and was more likely to have harmful alcohol consumption based on AUDIT scores (*p* = 0.039) than the HC group. Also, the case group had fewer years of education (χ2 = 33.16, df = 3, *p* < 0.001) showing that 54.68% of case participants had less than 12 years of study compared to 8.19% in the HC group. Sociodemographic characteristics were not evaluated in the follow-up visit.

**TABLE 1:** Descriptive participants’ characteristics.

Variables	HC group	Case group	*t*-test	*p*-value
Age (mean, standard deviation–SD)	28.13 (7.365)	24.3 (6.616)	3.065	0.003
Per-person income (mean, SD)	2,259.56 (2,658.83)	1,001.62 (1,064.36)	3.223	0.002
AUDIT score (mean, SD)	2.62 (2.34)	3.89 (4.22)	−2.092	0.039

*Age in years. Per-person income in BRL. AUDIT: Alcohol Use Disorders Identification Test.*

**FIGURE 1:**
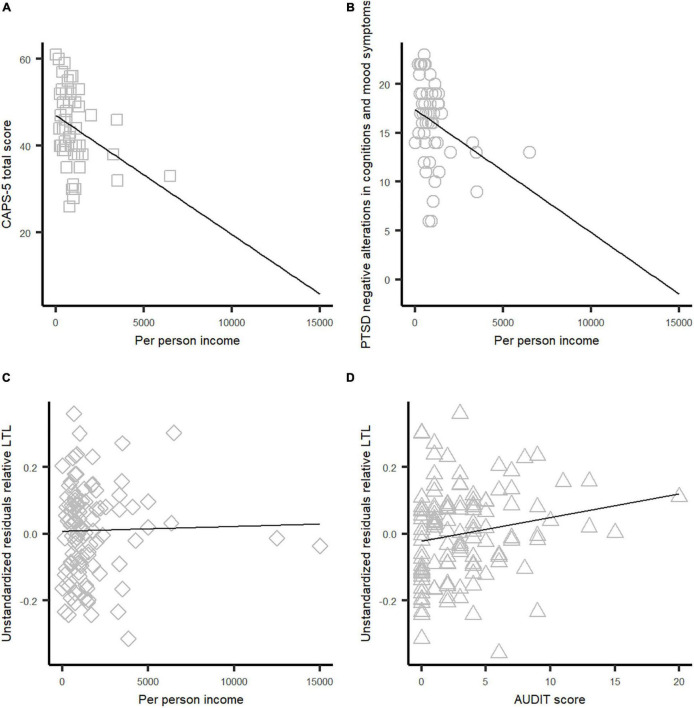
Scatter plots of correlations tests among per-person income, PTSD symptoms, and relative LTL adjusted by age. **(A)** Correlation between CAPS-5 total score and per-person income. **(B)** Correlation between negative alterations in cognitions and mood score and per-person income. **(C)** Correlation between relative LTL and per-person income. **(D)** Correlation between relative LTL and AUDIT score.

Correlation tests using baseline measures showed that per-person income was significantly inversely correlated with CAPS-5 total score (*p* = 0.003) (Figure 1A and Table 2) and negative alterations in cognitions and mood (*p* = 0.007) (Figure 1B and Table 2), whereas avoidance, re-experiencing, and hyperarousal symptoms did not correlate with per-person income (*p* > 0.05; Table 2). Furthermore, we observed that per-person income was positively correlated with relative LTL adjusted by age (*p* = 0.014) (Figure 1C and Table 2). Likewise, alcohol consumption was positively correlated with relative LTL (*p* = 0.045) (Figure 1D and Table 2). We did not find a significant correlation between per-person income and alcohol consumption measured by AUDIT score (Table 2).

**TABLE 2:** Correlation results of PTSD symptom clusters with per-person income, relative LTL adjusted by age and per-person income or alcohol consumption, and per-person income or alcohol consumption.

Correlations tests	Correlation coefficient	*p*-value
Per-person income x CAPS-5 total	Spearman rho = −0.406	**0.003**
Per-person income x Avoidance symptoms	Spearman rho = −0.234	0.092
Per-person income x Re-experiencing symptoms	Spearman rho = −0.187	0.180
Per-person income x Negative alterations in cognitions and mood symptoms	Spearman rho = −0.365	**0.007**
Per-person income x Hyperarousal symptoms	Spearman rho = −0.194	0.164
Per-person income x AUDIT score (harmful alcohol use)	Spearman rho = 0.010	0.944
Per-person income x Relative LTL adjusted by age (case group)	Pearson correlation = 0.338	**0.014**
Per-person income x Relative LTL adjusted by age (HC group)	Pearson correlation = −0.094	0.502
AUDIT score (harmful alcohol use) x Relative LTL adjusted by age	Pearson correlation = 0.182	**0.045**

*Bold values represent the significant p-values.*

### Leukocyte Telomere Length Findings

#### Baseline Leukocyte Telomere Length Measurements

The mean of baseline relative LTL measured in 63 patients with PTSD was 1.07 (standard deviation (SD) = 0.13), whereas the mean of LTL in 60 HC subjects was 1.09 (SD = 0.13). Furthermore, we did not find an association of shorter LTL with increasing age (*p* = 0.331). The Shapiro–Wilk test showed that LTL data were normally distributed (SW_baseline_ *p* = 0.70).

We verified that baseline relative LTL adjusted by age was not associated with trauma exposure (i.e., sexual assault) (*p* = 0.81). Further, we observed that the discrimination between case and HC groups based on relative LTL adjusted by age was not significant (*p* = 0.71), even adjusting the model for confounder effects: alcohol dependence, educational attainment, and per-person income.

**FIGURE 2:**
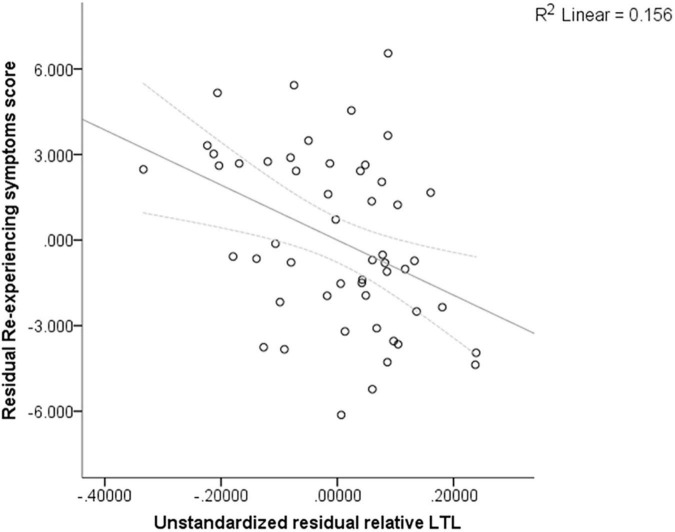
Partial regression plot for the linear regression adjusted by per-person income reflecting the correlation coefficient between PTSD re-experiencing symptoms and relative LTL adjusted by age.

We observed that PTSD participants with higher PTSD re-experiencing symptoms scores at baseline had shorter relative LTL adjusted by age (*B* = −0.016, 95% confidence interval: −0.027 to −0.005; Bonferroni-adjusted *p* = 0.02, power = 0.79), suggesting that for each unit increase in PTSD re-experiencing scores, a 0.016 decrease in relative LTL is expected (Figure 2). Nonetheless, after Bonferroni correction for multiple comparisons, no significant difference in relative LTL was identified for the other PTSD symptom clusters and for CAPS_5 total score (CAPS-5 total → adjusted *p* = 0.66, avoidance → adjusted *p* = 1.045, negative alterations in cognitions and mood → adjusted *p* = 1.2, hyperarousal → adjusted *p* = 3.24).

#### Follow-Up Leukocyte Telomere Length Measurements

At 1-year follow-up, for the three groups evaluated (PTSD persistent (*n* = 13), PTSD remitter (*n* = 11), and HC (*n* = 11)), there was a trend for shorter relative LTL in the PTSD remitter group compared to other groups; however, this mean difference did not significantly different among the groups (*p* > 0.05; PTSD remitter: average = 1.09 (SD = 0.03), PTSD persistent: average = 1.13 (SD = 0.03), HC: average = 1.32 (SD = 0.03)). Also, we did not observe any association of shorter LTL with increasing age (*p* = 0.531).

In addition, the Shapiro–Wilk test showed that the distribution of follow-up LTL data was normally distributed (SW_follow–up_ = 0.35) among groups.

In the longitudinal study, telomere shortening was no longer observed in PTSD participants. Linear regression did not reveal any significant associations between PTSD cluster symptoms (CAPS-5 total, avoidance, re-experiencing, negative alterations in cognitions and mood, and hyperarousal) and relative LTL adjusted by age (*p* > 0.05).

## Discussion

This study used cross-sectional and longitudinal datasets to investigate whether PTSD psychopathology (diagnosis and symptoms) was associated with shorter LTL in a Brazilian population of sexually assaulted young women compared to the HC group, after accounting for the effects of socioeconomic deprivation and harmful alcohol use. These analyses revealed a small association between shorter baseline relative LTL and higher re-experiencing symptoms measured at baseline, which partially supports our hypothesis that PTSD symptoms may decrease LTL. However, the longitudinal association between re-experiencing symptoms measured at follow-up (persistent PTSD and PTSD remitter) could not explain the variation in LTL among the groups investigated at baseline. Further, no significant associations between trauma exposure (i.e., sexual assault), PTSD diagnosis, and three clusters of PTSD symptoms (avoidance, hyperarousal, and negative alterations in cognitions and mood) and LTL were detected both at baseline and at follow-up.

We found significantly lower per-person income and educational attainment in the case group compared to HC group, as described in the literature. Previous studies have shown that individuals with low income are more exposed to stressful and more violent environments, possibly increasing exposure to trauma and subsequent risk of developing PTSD (34–36). Also, low educational attainment has been considered a risk factor for PTSD (37, 38), suggesting that individuals with higher educational levels exposed to trauma have more coping mechanisms to deal with posttraumatic consequences, perhaps due to more access to information and treatment options (38, 39).

We observed that participants with PTSD were more likely to present harmful alcohol use. Research findings report that alcohol use may function as a maladaptive coping behavior to mitigate PTSD symptoms (40, 41). Conversely, it may hamper PTSD recovery and indicate unfavorable psychopathological outcomes (42, 43). Further, the effects of increased alcohol drinking were previously associated with shorter TL (20–23) in patients with PTSD, corroborating our results. Our findings suggest that the combination of posttraumatic stress, socioeconomic deprivation, and harmful alcohol use may potentially lead to telomere erosion *via* alterations in the HPA axis responsiveness.

Telomere length is a complex phenotype related to genetic and environmental factors that may act as an important marker of stress and PTSD (19, 44). Telomere shortening observed in male war veterans (6, 10, 15) and women exposed to severely stressful events (16), including rape (17), has been associated with PTSD diagnosis. However, the results from published studies are mixed. Contrary to previous research (17), our study found that women with PTSD did not show differences in LTL compared to HC individuals. Our findings suggest that accelerated biological aging as measured through LTL may not be noticeable in young women with PTSD.

Recently, lifetime PTSD symptoms were associated with shorter TL in men and women (12, 15). However, no study investigated the link between TL and PTSD symptom clusters. To the best of our knowledge, this is the first study to show an association between PTSD re-experiencing symptoms and shorter LTL. Re-experiencing symptoms are the most characteristic symptoms of PTSD, denoted by intensive memories of the traumatic event, flashbacks, nightmares, and frightening thoughts (45). We suggest that PTSD re-experiencing symptoms may significantly increase the stress response system and exacerbate dysregulation of the HPA axis, leading to the elevation of concentrations of corticosteroids and immune system alterations accelerating telomere shortening (2, 5, 18, 19, 46–48).

Previous genetic association studies and polygenic risk scores (49–51) showed that re-experiencing symptoms have been associated with the *CRHR1* gene (corticotropin-releasing hormone receptor 1). *CRHR1* is involved in the stress-response system by allostatic load and immune response to stress (52–54). These studies provide additional evidence to our findings suggesting that re-experiencing symptoms may be related to impairment in regulating the immune system, which may cause TL changes. However, the literature lacks studies that investigated the association between PTSD re-experiencing symptoms and TL.

Despite the increasing number of evidence suggesting that re-experiencing and hyperarousal may be linked to biological mechanisms underlying PTSD status, our findings showed no association between hyperarousal symptom severity and LTL, contrary to our hypothesis. Previous studies have shown that hyperarousal symptoms may alter the HPA axis and brain reactivity (55–58); however, these studies have not been able to examine how chronic arousal may influence telomere shortening. Further studies with larger samples are needed to evaluate whether hyperarousal symptoms are linked to mechanisms underlying cellular aging.

Longitudinal analyses revealed no association between telomere shortening and PTSD diagnosis or PTSD symptom severity (avoidance, re-experiencing, hyperarousal, and negative alterations in cognitions and mood) in 24 women who completed the 1-year follow-up. It is important to emphasize that our longitudinal findings were not able to replicate the cross-sectional relationship between shorter relative LTL and higher re-experiencing symptoms. One possible explanation for our no significant findings is perhaps the low statistical power of our longitudinal analysis, as in genetic studies, small biological changes may not be detectable in small sample sizes. Also, understanding the longitudinal changes in TL remains challenging, as there is no evidence of how trauma or PTSD may contribute to TL maintenance.

The present findings must be interpreted in the context of some limitations. First, our study has a relatively small sample size. This may have reduced the statistical power to identify the minor effects of predictor variables in TL. Second, the assessment of TL by qPCR in leukocyte cells depends on the number of leukocytes in the blood, which may be affected by numerous factors, e.g., lifestyle and diseases. Thus, this method gives a relative mean of telomere attrition, i.e., providing information about the shortening rate. Third, the beta coefficient for predicting PTSD re-experiencing symptoms by LTL is relatively small; thus, we need caution to interpret these findings. Fourth, telomere shortening may be affected by genetic (i.e., genetic variants, gene expression) and environmental factors (i.e., lifestyle, smoking behavior, diet), and we were not able to adjust LTL for all variables, as we did not have them available in this study. Last, our analyses are limited to two time points (baseline and 1-year follow-up). Detecting accelerated biological aging may prove difficult after a 1-year period. Further multi-wave longitudinal studies are warranted to verify the effect of PTSD on LTL.

This study provides evidence that shorter baseline LTL may be related to the early stage of PTSD re-experiencing symptoms in recently sexually assaulted women. However, at the 1-year follow-up, we did not observe telomere shortening in both remitters and persistent patients with PTSD. Further, our findings did not replicate the previous association between PTSD diagnosis or trauma exposure and shorter TL. This study also suggests the potential effect of lower per-person income and harmful alcohol use on LTL. Future research should investigate underlying biological mechanisms which interplay TL, trauma, and PTSD, e.g., immune, and endocrine systems.

## Data Availability Statement

The original contributions presented in the study are included in the article, further inquiries can be directed to the corresponding author.

## Ethics Statement

The studies involving human participants were reviewed and approved by the Research Ethics Committee of Universidade Federal de São Paulo (UNIFESP). The patients/participants provided their written informed consent to participate in this study.

## Author Contributions

CMC and SIB participated in the conception and design of this study. CMC drafted the article. CMC, BMC, AFM, VKO, MFM, and SIB participated in data collection, data analysis, and data interpretation. GX, AVGB, TF, MO, and RP aided in the data interpretation. BMC, GX, AVGB, TF, MO, RP, AFM, VKO, MFM, and SIB made critical revisions to this article and agreed on final article before submission. All authors contributed to the article and approved the submitted version.

## Conflict of Interest

RP is paid for their editorial work on the journal Complex Psychiatry. The remaining authors declare that the research was conducted in the absence of any commercial or financial relationships that could be construed as a potential conflict of interest.

## Publisher’s Note

All claims expressed in this article are solely those of the authors and do not necessarily represent those of their affiliated organizations, or those of the publisher, the editors and the reviewers. Any product that may be evaluated in this article, or claim that may be made by its manufacturer, is not guaranteed or endorsed by the publisher.
